# ASSESSING MINDFULNESS FACETS TO INTERROGATE THE “WELL-BEING PARADOX” IN NIGERIA

**DOI:** 10.1093/geroni/igae098.1993

**Published:** 2024-12-31

**Authors:** Peace Amanambu, Eric Allard

**Affiliations:** University of Nigeria, Nsukka, Enugu, Nigeria; Cleveland State University, Cleveland, Ohio, United States

## Abstract

The role of mindfulness for explaining the well-being paradox in aging has been examined within Western samples. However, whether this association exists cross-culturally has not been previously addressed. Furthermore, investigations on the role of mindfulness facets on the associations between age and well-being is limited. The present study assessed whether distinct mindfulness facets (i.e., observing, describing, acting with awareness, non-judgment, and non-reactivity) mediate the relationship between age and positive and negative affect in an adult lifespan sample in Nigeria. Participants were 242 (95 women, age range = 18-75 years, Mage = 41.79; SDage = 13.58) adults, affiliated with the University of Nigeria-Nsukka, who completed the Positive and Negative Affect Scale and Five Facet Mindfulness Questionnaire (FFMQ). Increased age was associated with diminished negative affect (NA) but unrelated to positive affect (PA). In terms of mindfulness facets, older age was significantly related to increased endorsement of the Describing, Acting with Awareness, and Non-Reactivity facets. Separate mediation models were conducted with these three facets in predicting NA. Here, diminished age-related NA was mediated by the Describing facet. No significant mediating relationships were observed for Acting with Awareness and Non-Reactivity. The present findings suggest some cross-cultural coherence in mindfulness as an arbiter of the well-being paradox in aging, further specifying aspects of the mindfulness construct contributing to this relationship.

